# An Ultrasound Study of Cerebral Venous Drainage after Internal Jugular Vein Catheterization

**DOI:** 10.1155/2012/685481

**Published:** 2012-05-17

**Authors:** Davide Vailati, Massimo Lamperti, Matteo Subert, Alberto Sommariva

**Affiliations:** Department of Neuroanesthesia, Neurological Institute “C. Besta”, 20133 Milan, Italy

## Abstract

*Objectives*. It has been advocated that internal jugular vein (IJV) cannulation in patients at risk for intracranial hypertension could impair cerebral venous return. Aim of this study was to demonstrate that ultrasound-guided IJV cannulation in elective neurosurgical patients is safe and does not impair cerebral venous return. *Methods*. IJV cross-sectional diameter and flow were measured using two-dimensional ultrasound and Doppler function bilaterally before and after IJV cannulation with the head supine and elevated at 30°. *Results*. Fifty patients with intracranial lesions at risk for intracranial hypertension were enrolled in this observational prospective study. IJV diameters before and after ultrasound-guided cannulation were not statistically different during supine or head-up position and the absolute variation of the venous flow revealed an average reduction of the venous flow after cannulation without a significant reduction of the venous flow rate after cannulation. *Conclusions*. Ultrasound-guided IJV cannulation in neurosurgical patients at risk for intracranial hypertension does not impair significantly jugular venous flow and indirectly cerebral venous return.

## 1. Introduction

Patients with head injury, cerebral haemorrhage, brain tumors, and hydrocephalus have a hemodynamic that could be easily impaired. In these patients, internal jugular vein (IJV) represents the main cerebral venous output and any reduction in its flow could create an increase in cerebral blood volume and intracranial pressure (ICP) [1–3]. 

It has been advocated for decades that internal jugular vein cannulation should have to be avoided in any kind of neurosurgical patients in order to avoid intracranial hypertension (HICP) and subclavian vein cannulation was advised as the best choice even if this procedure could be associated with major and life-threatening complications. 

Furthermore, some authors demonstrated that the positioning of a central venous catheter (CVC) in the internal jugular vein may cause a lesion of the valve of the IJV [4] and a jugular vein incompetence [5] and there are some other manoeuvres that can cause this impairment till to the transient global amnesia [6, 7], with the appearance that a retrograde jugular flow is the cause of cardiovascular and neurological problems [8].

Several publications supported these assertions [9–12], while only two works stated the opposite. Goetting et al. [13] analyzed a population of 37 children with elevated ICP; after a central venous line placement in the IJV, variation of ICP was measured and this study demonstrated that IJV cannulation did not increase ICP. Woda et al. [14] took into consideration 11 adult patients, stating that the ICP increase after the positioning of CVC in IJV was not significant. These two studies did not evaluate the physiopathological compensatory mechanism that avoided cerebral venous output impairment.

When cerebral veins are suddenly blocked, the brain begins to undergo an engorgement process. By increasing the cerebral venous volume, the cerebrospinal fluid is reabsorbed and/or moved towards the subarachnoid space causing a reduction of the ventricles size. In order to restore normal values of pressure and volume of the cerebral venous blood, it causes a considerable effort in channeling the blood through the collateral vessels [15]. 

This response is definitely less valid in case of occlusion or acute obstruction of the main cerebral venous output, while it is more effective both in cases where thrombosis occurs slowly (e.g., invasion of the sagittal sinus by meningiomas) and in those where the obstruction is extracranial, using forms of cardio circulatory compensation. When major cerebral venous occlusion occurs, the brain is congested and interstitial oedema and haemorrhage could appear. Internal jugular vein cannulation may have a double effect on intracranial pressure. 

First, cerebral venous blood moves through the cerebral venous sinus, reaching the sigmoidal sinus, which drains in the jugular bulb and then in the internal jugular vein [1]. An occlusion, even if partial, of the IJV, which represents the main cerebral venous draining system, could cause an engorgement of the venous sinus system with a consequent increase of ICP [2]. The second mechanism results in the inability of the cerebrospinal fluid to leave the skull through the arachnoid granulations.

The cerebral venous pressure is generally around 5 mm Hg, while the cerebrospinal fluid has pressure values between 5 and 20 mm Hg [16]. If the venous pressure increases due to the obstruction of the draining system, cerebrospinal fluid could not be removed, as what normally happens from the arachnoid villi. This series of events can cause an increase of ICP due to an increased amount of cerebrospinal fluid.

Ultrasound-guided cannulation has been suggested to be safe and effective by meta-analyses and guidelines [17–20]. For this reason this procedure should be preferred to subclavian cannulation in order to avoid post procedural major complications but with avoiding cerebral damage. Our primary endpoint was to measure, by means of ultrasound, if IJV cross-sectional diameter and flow in elective neurosurgical patients at risk for intracranial hypertension were different before and after IJV cannulation. Our secondary endpoint was to measure IJV before and after its cannulation when the head was placed in supine position and the head tilted up at 30°, a commonly used position for treatment of intracranial hypertension.

## 2. Materials and Methods

National Neurological Institute “C. Besta” Ethics Committee was informed according to Italian Guidelines for clinical observational studies and approved this study. Between November 2010 and May 2011, fifty patients affected by intracranial lesions, with neurological and radiological findings of intracranial hypertension (presence of two or more of the following signs: midline shift >1 cm, cerebral oedema, reduction of mesencephalic cisterns, obstructive hydrocephalus), were recruited. Inclusion criteria were patients scheduled for major neurosurgical procedures, ASA-physical status I and II, GCS 12–15 requiring a central venous line for perioperative hemodynamic management after informed written consent. 

Exclusion criteria were emergency surgery, ASA physical status 3 or more, any condition causing elevated right-sided pressures, GCS ≤ 10, any alteration of bleeding according to British Society of Haematology [18], previous neck surgery (thyroidectomy, tracheostomy, radical neck dissection), IJV thrombosis detected by compressive ultrasound [21], and patients with an accidental carotid artery puncture and/or a multiple vein puncture.

**Study Design**

**Figure 1:**
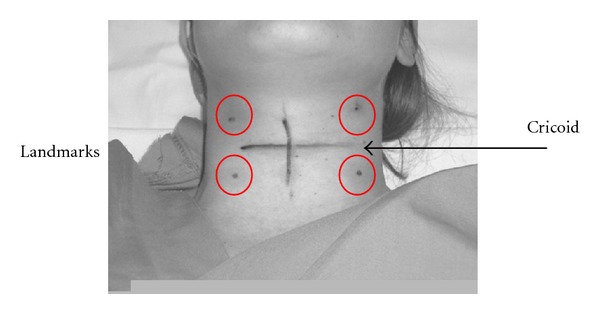
Landmarks used for measurements (in circle) and cricoid cartilage (arrow).

Five anaesthesiologists experts in ultrasound-guided cannulation and with advanced ultrasound skills performed the study. During the study, the same ultrasound machine (MicroMaxx, Sonosite Inc. Bothell, WA, USA) with a 13–6 MHz broadband linear probe (L25e, Sonosite Inc. Bothell, WA, USA) and Doppler function was used.Central venous cannulation of the IJV was performed using a double-lumen catheter (Arrow International, Reading, USA) avoiding occupying more than 1/3 of the IJV sectional diameter with the catheter (e.g., a 5 mm cross-sectional IJV diameter was occupied with a catheter no more than 5 F). All IJV catheterizations were done using a real-time US-guided technique with short-axis visualization of the vein an out-of-plane puncture.In all cases there were two anaesthesiologists performing the study. The first one was in charge of positioning the probe on the skin landmarks and of cannulating IJV; the second one was responsible for measuring sizes and flows of the jugular veins before and after CVC placement. All cannulation procedures were carried when patients were on general anaesthesia, mechanically ventilated, and in supine position. IJV measurements were performed at two points for each jugular vein at end expiration (Figure 1) [22]. Having identified the cricoid cartilage, two points were marked bilaterally: the first one located 2 cm up of the cricoid in correspondence with the IJV and the second one located 2 cm down of the cricoid in correspondence with the IJV. At this point bilateral IJV cross-sectional diameters, IJV cross-sectional area, velocimetry, and valve continence (by mean of Color-Doppler function) were measured. The same measurements were therefore carried out with patient's head in supine position (0 degree) and head tilted up at 30°. The head elevation was obtained and measured by protractor. With a sterile technique (neck skin disinfection and probe isolation with sterile cover) and under ultrasound guidance, IJV ultrasound-guided cannulation was then performed at a point between the two landmarks labelled. An out-of-plane technique was used for vein puncture. The choice for cannulating right or left IJV was taken after measuring IJV cross-sectional diameters and flows and according to surgical requirements. The larger IJV (dominant) was usually cannulated. After central venous line placement, the same measurements were repeated at same points previously marked, on both sides and with the head in supine position and tilted up at 30°.

**Data Collection**

Patient's demographic data, ASA-physical status, body-mass index, location, and type of intracranial lesions were recorded in a special data collection sheet. Ultrasound measurements were performed collecting major IJV cross-sectional transverse diameter, IJV cross-sectional area, and IJV Doppler velocimetry in the four-labelled landmarks points. IJV flow was calculated as result of the sum of the values at the top or at the bottom of the IJV (e.g., IJV top flow 0° = IJV right side flow 0°+ IJV left side flow 0°). All these measurements were repeated before and five minutes after IJV cannulation. Cerebral venous flow was calculated according to the formula:

(1) $$\begin{array}{cc} \text{Flow}\;\left(\text{mL}/\min\right) & =\text{IJV}\;\text{cross-sectional}\;\text{area}\;\left({\text{cm}}^{2}\right)\\ & \;\times \text{Doppler}\;\text{Velocimetry}\;\left(\text{cm/sec}\right)\times 60. \end{array}$$

Mean IJV flow variation rate was calculated according to the formula:

(2) $$\begin{array}{cc} \text{IJV}\;\text{Variation}\;\text{rate} & =(\text{IJV}\;\text{flow}\;\text{after}\;\text{cannulation}\;\;\;\\ & \;\;\;-\text{IJV}\;\text{flow}\;\text{before}\;\text{cannulation})\\ & \;\;\times 100/\text{IJV}\;\text{flow}\;\text{before}\;\text{cannulation}. \end{array}$$

In order to assess if IJV cannulation impaired cerebral venous output, the anaesthesiologist that performed the cannulation asked the neurosurgeon before dura mater opening to grade the rate of intracranial hypertension with a clinical subjective score (1-normal appearance of the dura mater, 2 thin dura mater, 3-brain swelling after dura mater opening).

**Statistical Analysis**

In order to calculate patients' sample size we hypothesised to detect an increase of mean IJV cross-sectional of 30% from 1.3 to 1.7 mm (SD 0.5) (*α* = 0.05; *β* = 0.1). For this purpose, we enrolled 50 patients. All data are presented as means and their standard deviations. A *t*-test for paired data was used. The mean diameters before and after central venous cannulation were compared using a *t*-test for paired data. The normality of flows and diameters distributions was evaluated using the Kolmogorov-Smirnov test. A *P*-value <0.05 was considered as significant.

## 3. Results and Discussion

Fifty patients were included in the study; four patients were excluded after IJV cannulation because of repeated vein puncture (*n* = 3) and one after multiple jugular puncture with concomitant accidental carotid puncture (*n* = 1; right and left IJVs were posterior to carotid artery). These major complications could probably be avoided if in-plane real-time ultrasound needle guidance would be used for IJV cannulation. Forty-six patients were successfully included in the analysis. Demographics' characteristics are depicted in Table 1. 

Diameters of the jugular veins measured in each landmark are expressed as mean ± standard deviation (Table 2). There was no significant difference between IJV cross-sectional diameters before and after cannulation, both at 0° and at 30° degree of head elevation.

Flow of the jugular vein measured at each landmark is expressed as mean ± standard deviation (Table 3). Ipsilateral and contra lateral IJV flows were not significantly different after cannulation. For this reason, we calculated IJV global flow and IJV global flow variation rate. A reduction of the absolute variation of IJV flows was observed at each landmark point, but with significant value only at the bottom point of the IJV when the head was at 0° (IJV 0° bottom: −68.2 (*P* = 0.01)). However, IJV flow variation rate resulted to be not significant in any of the four landmark points. 

At each measuring point, there was also a reduction of the mean values of the IJV flow when the head was tilted up from 0° to 30° both in the cannulated and the non cannulated vein.

Data obtained by Color-Doppler analysis of the IJV valve after IJV cannulation did not reveal any valve incontinence after central venous line placement. No intra operative clinical signs of intracranial hypertension (grade 2 or 3: thin dura, brain swelling) were recorded in these patients.

Our study demonstrated that IJV cannulation in elective neurosurgical patients at risk for intracranial hypertension does not impair cerebral venous return. In these patients, IJV diameters and venous flow were studied before and after central venous cannulation and with patients lying in supine position and with head tilted up to 30°. Our results demonstrate that, in supine position, mean IJV cross-sectional diameters at the top of the IJV were reduced after cannulation while they were increased at the bottom of the vein after cannulation. On the contrary, when the head was tilted up to 30°, IJV diameters increased at all points of examination after vein cannulation. Despite all these differences were not significant before and after cannulation, these differences suggest how the elasticity of the vein wall allows reestablishing a balance in the vein flow increasing IJV diameter and maintaining the same cerebral venous output. The mechanism of compensation should be an opening of the IJV valves that do allow impairing cerebral venous return.

IJV flow variation rates demonstrate a light reduction of the cerebral venous drainage after IJV cannulation. This, in part, could justify why all our patients did not have any intra-operative clinical signs of cerebral oedema. 

There are no previous data regarding IJV flow measurements in patients with intracranial hypertension or at risk for it because of intracranial masses. It has been suggested that a 30° tilting of the head could reduce cerebral blood volume by increasing cerebral venous drainage. 

Given our results it seems that this is not justified because after positioning the head at 30° there was a reduction of the IJV global flow both when IJV was free from catheter and when IJV was cannulated. We have no clear explanation for these data but one main concern could be that in our study we measured IJV flow only five minutes after tilting the head and after positioning the catheter. One more explanation could be that cerebral blood volume after head elevation is altered not only in terms of output (venous drainage) but also in input (arterial flow). Our measurements were done probably too early after head elevation and this could not be enough to allow a balance for the cerebral hemodynamic.

## 4. Conclusions

Central venous catheter placement in the IJV determines not significant changes in the cerebral venous return increasing mean IJV diameters and a reduction in mean IJV global flow in the internal jugular veins. Our results confirm that cerebral venous output has a good compensation system. IJV central venous cannulation is safe because it does not create any significant reduction in cerebral venous flow drainage in patients with risk for cerebral hypertension. The increase of IJV diameters demonstrates that there are some changes after central vein cannulation and an ultrasound evaluation of these diameters and IJV flow should be performed after IJV cannulation in every patient with intracranial hypertension or when bilateral cannulation of the internal jugular vein has to be performed (e.g., for jugular bulb oxygen saturation monitoring). 

Further studies are required in order to determine the cause of mean IJV flow reduction when the head is elevated at 30° by measuring both components of the cerebral blood volume and to evaluate if a longer time of head elevation allows cerebral flows to obtain a balance and a reduction of ICP.

Our study has some limits due to the small sample size and because intracranial pressure was not measured during IJV measurements by assessing it with a simple clinical score. Further research should be focused on a large population of patients with intracranial hypertension in order to determine if the dimensions of the central venous catheters impact on cerebral venous return. 

Ultrasound-guided cannulation of the IJV is a safe procedure and, when using ultrasound, a study of IJV diameters and flows in neurosurgical patients could avoid cannulating the vein with the wrong central venous catheter worsening cerebral damage.

## Figures and Tables

**Table 1:** Demographic characteristics. ^a^Values are expressed as mean ± SD.

	Total (*n* = 46)
Sex (M/F)	22/24
Age^a^ (years)	51.73 ± 14.30
BMI^a^	26.07 ± 5.24
ASA physical status (I/II)	21/25
IJV cannulation side (right/left)	32/14

**Table 2:** Analysis of IJV cross-sectional diameters for each landmark point, with head in supine and head elevation at 30° (mean ± SD).

		Top			Bottom		
		IJV diameter before cannulation	IJV diameter after cannulation	*P*-value	IJV diameter before cannulation	IJV diameter after cannulation	*P*-value
Head 0°	R	1.29 ± 0,38	1.23 ± 0.34	0.36	1.50 ± 0.57	1.55 ± 0.50	0.41
	L	1.03 ± 0.33	1.13 ± 0.43	0.09	1.21 ± 0.44	1.26 ± 0.46	0.29
							
Head 30°	R	0,95 ± 0,41	0,99 ± 0,30	0.47	1,14 ± 0,49	1,22 ± 0,48	0.14
	L	0,80 ± 0,27	0,85 ± 0,29	0.18	0,96 ± 0,40	0,97 ± 0,42	0.72

**Table 3:** Flows analysis results.

		IJV flow before cannulation	IJV flow after cannulation	Absolute variation		IJV flow variation rate	
		Mean ± SD	Mean ± SD	Mean	*P*-value*	Mean	*P*-value**
IJV 0°	Apex	891.4 ± 440.9	854.5 ± 396.1	−36.9	0.14	−1.8%	0.44
	Base	833.7 ± 384.3	765.5 ± 376.8	−68.2	0.01	−5.5%	0.14
IJV 30°	Apex	540.8 ± 375.1	515.7 ± 347.4	−25.1	0.12	−2.7%	0.31
	Base	481.8 ± 315.5	465.4 ± 293.5	−26.4	0.18	−0.4%	0.90

## References

[1] Woodburne RT (1983). *Essentials of Human Anatomy*.

[2] Kaplan HA, Browder A, Browder J (1973). Narrow and atretic transverse dural sinuses: clinical significance. *Annals of Otology, Rhinology and Laryngology*.

[3] Langfitt TW, Tannanbaum HM, Kassell NF (1966). The etiology of acute brain swelling following experimental head injury.. *Journal of Neurosurgery*.

[4] Chung CP, Hsu HY, Chao AC, Wong WJ, Sheng WY, Hu HH (2007). Flow volume in the jugular vein and related hemodynamics in the branches of the jugular vein. *Ultrasound in Medicine and Biology*.

[5] Wu X, Studer W, Erb T, Skarvan K, Seeberger MD (2000). Competence of the internal jugular vein valve is damaged by cannulation and catheterization of the internal jugular vein. *Anesthesiology*.

[6] Lewis SL (1998). Aetiology of transient global amnesia. *Lancet*.

[7] Chung CP, Hsu HY, Chao AC, Chang FC, Sheng WY, Hu HH (2006). Detection of intracranial venous reflux in patients of transient global amnesia. *Neurology*.

[8] D’Cruz IA, Khouzam RN, Minderman DP, Munir A (2006). Incompetence of the internal jugular venous valve: spectrum of echo-Doppler appearances. *Echocardiography*.

[9] McGee WT, Mallory DL (1988). Cannulation of the internal and external jugular veins. *Problems in Critical Care*.

[10] Rubenstein JS, Hageman JR (1988). Monitoring of critically ill infants and children. *Critical Care Clinics*.

[11] Venus B, Mallory DL, Civetta JM, Taylor RW, Kirby RR (1992). Vascular cannulation. *Critical Care*.

[12] Stocchetti N, Longhi L, Valeriani V (2003). Bilateral cannulation of internal jugular veins may worsen intracranial hypertension. *Anesthesiology*.

[13] Goetting MG, Preston G (1991). Jugular bulb catheterization does not increase intracranial pressure. *Intensive Care Medicine*.

[14] Woda RP, Miner ME, McCandless C, McSweeney TD (1996). The effect of right internal jugular vein cannulation on intracranial pressure. *Journal of Neurosurgical Anesthesiology*.

[15] Albin MS (1997). *Textbook of Neuroanesthesia with Neurosurgical and Neuroscience Perspectives*.

[16] Daube JR, Sandok BA (1978). *Medical Neurosciences*.

[17] National Institute for Clinical Excellence Guidance on the use of ultrasound locating devices for placing central venous catheters. http://www.nice.org.uk/.

[18] Bishop L, Dougherty L, Bodenham A (2007). Guidelines on the insertion and management of central venous access devices in adults. *International Journal of Laboratory Hematology*.

[19] Troianos C, Hartman G, Glas K (2011). Guidelines for performing ultrasound guided vascular cannulation:
recommendations of the american society of echocardiography and
the society of cardiovascular anesthesiologists. *Journal of the American Society of Echocardiography*.

[20] Randolph AG, Cook DJ, Gonzales CA, Pribble CG (1996). Ultrasound guidance for placement of central venous catheters: a meta-analysis of the literature. *Critical Care Medicine*.

[21] Kory PD, Pellecchia CM, Shiloh AL, Mayo PH, DiBello C, Koenig S (2011). Accuracy of ultrasonography performed by critical care physicians for the diagnosis of DVT. *Chest*.

[22] Takeyama K, Kobayashi H, Suzuki T (2005). Optimal puncture site of the right internal jugular vein after laryngeal mask airway placement. *Anesthesiology*.

